# Chromosome 15 structural abnormalities: effect on *IGF1R* gene expression and function

**DOI:** 10.1530/EC-17-0158

**Published:** 2017-08-18

**Authors:** Rossella Cannarella, Teresa Mattina, Rosita A Condorelli, Laura M Mongioì, Giuseppe Pandini, Sandro La Vignera, Aldo E Calogero

**Affiliations:** 1 Department of Clinical and Experimental MedicineUniversity of Catania, Catania, Italy; 2 GeneticsUniversity of Catania, Catania, Italy

**Keywords:** IGF1, IGF1R, cryptorchidism

## Abstract

Insulin-like growth factor 1 receptor (*IGF1R*), mapping on the 15q26.3 chromosome, is required for normal embryonic and postnatal growth. The aim of the present study was to evaluate the *IGF1R* gene expression and function in three unrelated patients with chromosome 15 structural abnormalities. We report two male patients with the smallest 15q26.3 chromosome duplication described so far, and a female patient with ring chromosome 15 syndrome. Patient one, with a 568 kb pure duplication, had overgrowth, developmental delay, mental and psychomotor retardation, obesity, cryptorchidism, borderline low testis volume, severe oligoasthenoteratozoospermia and gynecomastia. We found a 1.8-fold increase in the IGF1R mRNA and a 1.3-fold increase in the IGF1R protein expression (*P* < 0.05). Patient two, with a 650 kb impure duplication, showed overgrowth, developmental delay, mild mental retardation, precocious puberty, low testicular volume and severe oligoasthenoteratozoospermia. The IGF1R mRNA and protein expression was similar to that of the control. Patient three, with a 46,XX r(15) (p10q26.2) karyotype, displayed intrauterine growth retardation, developmental delay, mental and psychomotor retardation. We found a <0.5-fold decrease in the IGF1R mRNA expression and an undetectable IGF1R activity. After reviewing the previously 96 published cases of chromosome 15q duplication, we found that neurological disorders, congenital cardiac defects, typical facial traits and gonadal abnormalities are the prominent features in patients with chromosome 15q duplication. Interestingly, patients with 15q deletion syndrome display similar features. We speculate that both the increased and decreased *IGF1R* gene expression may play a role in the etiology of neurological and gonadal disorders.

## Introduction

Insulin-like growth factor 1 receptor (*IGF1R*) gene, made up of 315,991 base pairs, maps on the 15q26.3 chromosome. It encodes for a protein with a tyrosine kinase domain, which binds the IGF1 and is responsible for its biological activity. Chromosomal 15q structural abnormalities, such as distal duplication or ring 15, can alter the *IGF1R* expression and function.

Up to now, more than seventy patients with chromosome 15q terminal duplication have been reported (1). The majority of them have a terminal duplication with a proximal breakpoint ranging from 15q25.1 or 15q26.1 to the terminus (2). This structural chromosomal abnormality causes a common phenotype that includes prenatal and postnatal overgrowth, intellectual disability, characteristic craniofacial dimorphism and renal abnormalities (3), resulting in the so-called 15q overgrowth syndrome. However, failure to thrive and/or intrauterine growth retardation (IUGR) have been reported in some patients with chromosome 15q terminal duplication (2, 4, 5), thus suggesting that triplication of the *IGF1R* gene does not seem to be sufficient to cause somatic overgrowth.

Patients with ring chromosome 15 have been associated with growth delay, microcephaly, triangular face and a variable degree of mental retardation (6). Approximately 40 cases have been reported in literature (7).

The aim of the present study was to evaluate the IGF1R mRNA and protein expression and the IGF1R protein activity in two male patients with chromosome 15q duplication and in one female patient with ring chromosome 15. The phenotypes of these three patients have been framed in the context of those found in the other published cases by reviewing the literature.

## Patients and methods

### Case report

#### Patient one

Patient 1 was a 19-year-old male, who had attended the endocrinology outpatient clinic since the age of 14 years, complaining from tall stature. His birth weight was 3150 g and he had a left undescended testis, that was rescued by hCG administration for 6 weeks when he was 2 years old. He was also diagnosed for a moderate mental and psychomotor retardation, crossness, uneasiness and he had a marked defective speech capacity. The cardiologic counseling revealed no abnormality.

On physical examination, he had high-arched palate, ptosis and scoliosis. At the age of 8 years, he weighed 56.2 kg (>97th percentile), he was 144.9 cm (>99th percentile) tall and had a cranial circumference (CC) that measured 56 cm (>97th percentile). At the age of 14 years, these measurements were, respectively, 97 kg (>97th percentile), 176 cm (90th percentile) and 57.5 cm (>97th percentile). The testicular volume (TV) was 4 mL bilaterally and both testes had an increased consistency. Basal serum luteotropin hormone (LH), follicle-stimulating hormone (FSH) and testosterone (T) levels were 5.61 IU/mL (normal values (n.v.): 1.5–9.3 IU/mL), 5.37 IU/mL (n.v.: 1.4–18.1 IU/mL) and 1.87 ng/mL (n.v.: 3–9 ng/mL), respectively. When he was 15 years old, the TV measured 8 mL bilaterally, and the T levels were 2.9 ng/mL. When he was 17 years old, he was at Tanner stage 3, he had a triangular distribution of pubic hair and a bilateral TV of 12 mL. Serum LH, FSH and T levels were, in turn, 4.8 IU/mL, 9.1 IU/mL and 3.96 ng/mL, and the IGF1 levels were 411.3 ng/mL (n.v.: 119–395 ng/mL). He was 180 cm (50th–75th percentile; target height: 166.5 cm) tall. At the age of 19 years, the sperm analysis detected a severe oligoasthenoteratozoospermia (total sperm count 0.5 million/ejaculate – n.v. >39 million/ejaculate, forward sperm motility 3% – n.v. >32%, total sperm motility 10% – n.v. >40%, normally shaped spermatozoa 2% – n.v. >4%).

Genetic analysis excluded both Prader–Willi and Kallmann syndrome (KAL1, FGFR1, PROK2, PROK2R were evaluated and no mutations resulted). He had a 46,XY karyotype and a 568 kb pure duplication on the 15q26.3 chromosome, diagnosed by array-CGH. This duplication was not detected in the mother. The father could not be evaluated because he was not alive. The patient sister had a normal psychophysical development, and she did not show overgrowth.

#### Patient two

Patient 2 was a 16-year-old male, admitted for the first time to the Department of Pediatrics, teaching hospital ‘G. Rodolico’, University of Catania, at the age of 7 years, for clinical signs of precocious puberty. He had a marked defective speech capacity, a low-grade mental retardation (IQ 66), a benign tachycardia and a low-grade insufficiency of pulmonary valve. The abdomen ultrasound, the brain magnetic resonance and the electroencephalogram revealed no abnormality.

On physical examination, performed at the age of 7 years, he weighed 39.8 kg (97th percentile), and he was 147.5 cm (>97th percentile) tall. The CC measured 54 cm (75th percentile). Seven café-au-lait spots were detected, the larger (7 × 4 cm) in the right groin, the others in the right clavicular region, left lower limb, left hip and right gluteus. He was at Tanner stage 3, and both testes had a volume of 8 mL. His serum T levels were higher for his age; the bone age was advanced by two years. The GnRH analog test showed results compatible with precocious puberty (serum hormone levels not available). On this basis, he was prescribed Gonapeptyl Depot (1 injection every 28 days), which was administered from the age of 8 to that of 11 years.

At the age of 11 years, serum IGF1 levels were in the normal range (262.8 ng/mL; n.v.: 49–520 ng/mL). At the age of 14 years, he weighed 77 kg (95th percentile) and he was 171 cm (75th percentile) tall. He was at Tanner stage 5 and his LH and T serum levels were, respectively, 3.18 IU/mL and 3.74 ng/mL. At the age of 16 years, the TV was 11 mL, bilaterally, and the LH, FSH and T values were, in turn, 2.93 IU/mL, 1.94 IU/mL and 5.26 ng/mL. The sperm analysis detected a severe oligoastenotheratozoospermia (total sperm count 0.15 million/ejaculate, forward motility 3%, total motility 13%, normally shaped spermatozoa 2%).

The genetic analysis showed a 46,XY karyotype with a 650 kb impure duplication on the 15q26.3 chromosome (a 600 kb deletion on the 16p11.2 chromosome was also found). The NF-1 (exons 1–58), OMG (exon 2), ASPA (exon 6), PMP22 (exon 3), TRAF4 (exon 2, 4), SSH2 (exon 4, 14), BLMH (exon 2), CPD (exon 12), SUZ12P (exon 1, 3), CRLF3 (exon 3), ATAD5 (exon 2), ADAP2 (exon 3), RNF 135 (exon 2), UTP6 (exon 14), SUZ12 (exon 10), LRRC37B (exon 1), ZNF207 (exon 9), PSMD11 (exon 2, 6) and MYO1D (exon 2, 7) genes were evaluated to exclude the presence of neurofibromatosis. The genetic testing revealed no abnormality. The clinical history of his biological parents is unknown since he was adopted.

#### Patient three

Patient 3 was a 6-year-old female patient presented to the Department of Pediatrics, teaching hospital ‘G. Rodolico’, University of Catania, at the age of 6 years, complaining from developmental delay. Her birth weight was 1860 g, and her length was 44 cm. She had mental and psychomotor retardation.

On physical examination, she weighed 10.3 kg (<3rd percentile), she was 92 cm (<3rd percentile) tall and the CC measured 41 cm (<3rd percentile). Café-au-lait spots in the left side of chest, in the right leg, in the groin and vitiligo in the right side of chest were detected. The diagnosis of neurofibromatosis was clinically excluded. She had triangular face, microcephaly, thin hairs, arched eyebrows, blepharophimosis, broad nasal bridge and slight superior lip. She had clinodactyly and shortness of the second finger. The X-ray showed a bilateral extra phalanx in the third finger, a bilateral hypoplasia of the phalanx of the third finger and a bilateral dysmorphic and hypoplastic phalanx in the second finger. FISH analysis detected a 46,XX r(15) (p10q26.2) karyotype. According to array-CGH results, she had a deletion of the last 5 Mb of chromosome 15 and a duplication of 2 Mb. An absence of paternal alleles in the terminal 15q chromosome (from 95.258 Mb to qter) was found at the microsatellites analysis. Thus, she did not have a paternal origin of the rearrangement. At the age of 11 years, serum IGF1 levels were 186 ng/mL (n.v.: 87.4–399.3 ng/mL). She underwent GH replacement therapy from the age of 14 years until the age of 16 years.

### Cell cultures

Lymphocytes were isolated from whole blood by Ficoll, according to the manufacturer’s instructions using Ficoll-Paque Plus (Amersham). The cells obtained with this procedure were grown for 48 h in RPMI medium supplemented with 10% FBS, 1% glutamine and 2 µg/mL phytohemagglutinin, divided in two aliquots: one for RNA extraction and one for protein analysis.

### Real-time PCR

Total RNA (5 µg) was reverse-transcribed by ThermoScript RT (Invitrogen) with Oligo dT primers. Synthesized cDNA (25 ng) was then used for a quantitative real-time PCR, using the following primers: 5′-GGG-CCA-TCA-GGA-TTG-AGA-AA-3′ (forward) and 5′-CAC-AGG-CCG-TGT-CGT-TGT-CA-3′ (reverse) specific for the *IGF1R* (fragment size, 330 bp) and 5′-ATT-GAA-GAA-ATT-GCA-GGC-TC-3′ (forward) and 5′-TGG-AGA-AGA-GGA-GCT-GTA-TCT-3′ (reverse) specific for the *ELE1* (housekeeping gene) (fragment size, 280 bp), on an ABI Prism 7500 (PE Applied Biosystems) using Sybr Green PCR Master Mix (PE Applied Biosystems) following manufacturer’s instructions. Amplification reactions were checked for the presence of nonspecific products by dissociation curve analysis. Relative quantitative determination of target gene levels was performed by comparing ΔCt, as described by Ginzinger. The PCR products were analyzed by 2% agarose gel electrophoresis and stained by Sybr Safe.

### IGF1R autophosphorylation

Lymphocytes cultured for 48 h were serum-starved for 24 h being cultured in serum-free medium before undergoing stimulation with 10 nM IGF1 for 5 min. Cells were lysed in cold RIPA buffer containing 50 mM Tris pH 7.4, 150 mM NaCl, 1% Triton X-100, 0.25% sodium deoxycolate, 10 mM sodium pyrophosphate, 1 mM NaF, 1 mM sodium orthovanadate, 2 mM PMSF, 10 µg/mL aprotinin, 10 µg/mL pepstatin, 10 µg/mL leupeptin and the insoluble material separated by centrifugation at 10,000 ***g*** for 15 min at 4°C. Cell lysates were subjected to SDS-PAGE and the resolved proteins were transferred to nitrocellulose membranes, immunoblotted with anti-phospho-IR/IGF1R (Tyr1158/Tyr1162/Tyr1163) antibody and detected by ECL. The nitrocellulose membrane was then stripped with buffer Restore (Pierce) and, subsequently, reprobed with an anti-IGF1R rabbit polyclonal antibody. The membranes were blotted with an anti β-actin antibody to control for protein loading.

### Statistical analysis

Results are expressed as mean ± s.e.m. The data were analyzed by one-way analysis of variance (ANOVA) followed by Bonferroni *post hoc* test. SPSS 22.0 for Windows was used for statistical analysis (SPSS). Statistical significance was accepted when the *P* value was lower than 0.05.

Consent has been obtained from each patient or subject after full explanation of the purpose and nature of all procedures used. An approval by an ethical Committee was not required because the data presented were obtained during the routine diagnostic workout, which the three patients underwent to within the Teaching Hospital of the University of Catania.

## Results

### IGF1R mRNA expression

**Figure 1:**
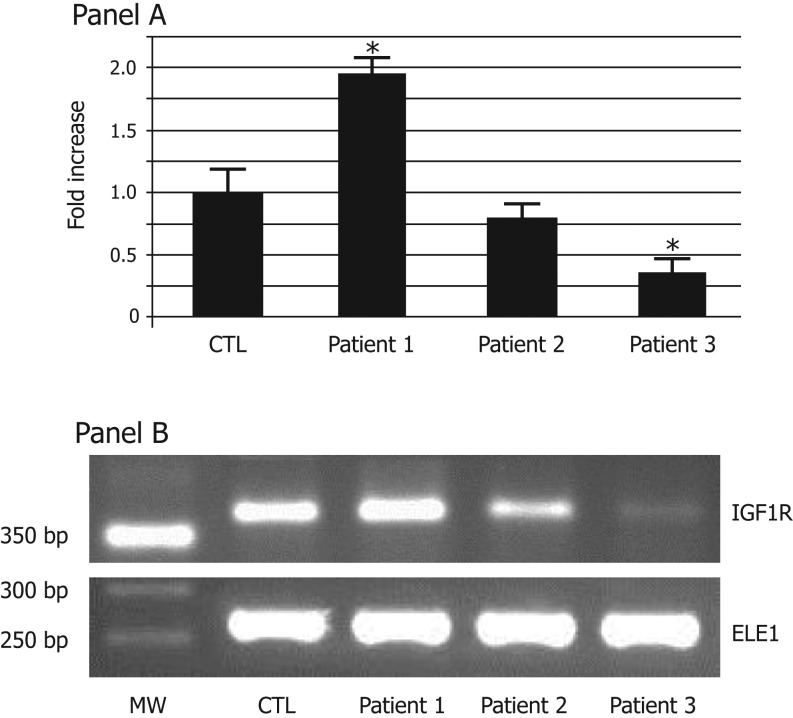
IGF1R mRNA expression. One microgram of RNA extracted from lymphocytes was used as template for real-time RT-PCR, as described in ‘Methods’ section. Panel A: IGF1R mRNA was evaluated by quantitative real-time PCR. Data were normalized with respect to ELE1 mRNA expression. Results are given as fold-changes of control (CTL). Data are the mean ± s.e.m. for two independent experiments. *P < 0.05. Panel B: The PCR product were analyzed in a 2% agarose gel electrophoresis and stained by SyBr Safe. CTL, control; MW, molecular weight; ELE, elongated empty glume (housekeeping gene).

The IGF1R mRNA expression, evaluated in lymphocytes using a relative quantification by real-time PCR, is shown in Fig. 1. A healthy 17-year-old male with a normal karyotype served as control. We found that the IGF1R mRNA expression was significantly higher (1.8 ± 0.25 fold) in Patient 1 compared to the average IGF1R mRNA expression of the control, whereas Patient 2 had a IGF1R mRNA expression similar to that found in the control. The IGF1R mRNA expression was significantly lower in Patient 3, resulting in a decrease <0.5-fold compared to that found in the control (Fig. 1A). Relative IGF1R mRNA expression was normalized to the abundance of ELE1 mRNA. Amplification reactions were checked for the presence of nonspecific products by agarose gel electrophoresis (Fig. 1B).

### IGF1R content and IGF1R tyrosine kinase activity in response to IGF1

**Figure 2:**
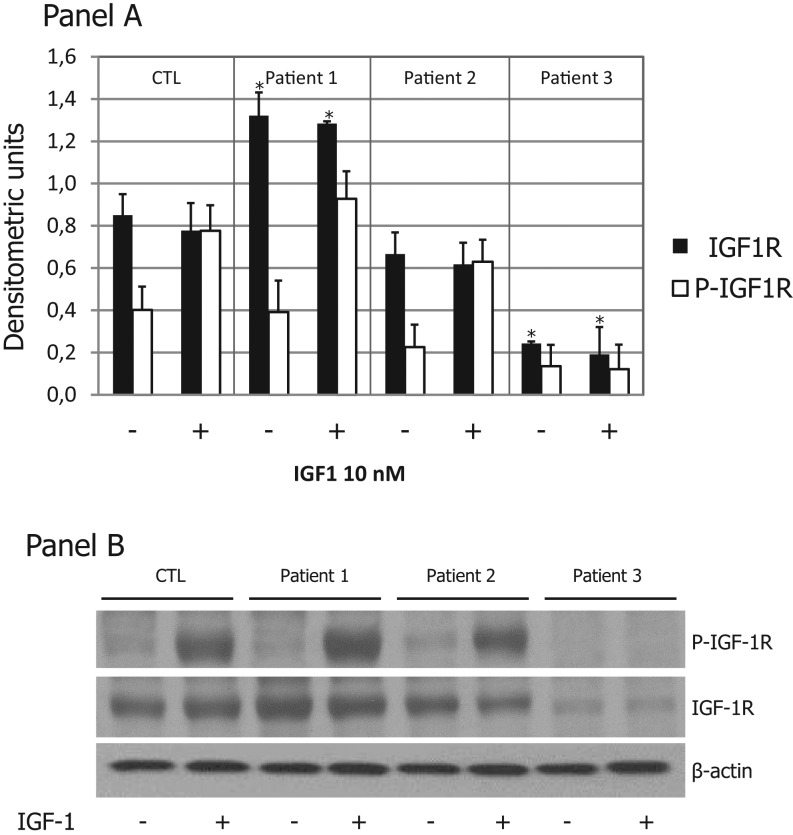
IGF1R autophosphorylation and IGF1R protein expression. Lymphocytes isolated as described in the ‘Materials and methods’ section were lysed in RIPA buffer. Panel A: Cell lysates underwent to SDS-PAGE and the resolved proteins were transferred to nitrocellulose membranes, immunoblotted with anti-phospho-1R/IGF1R (Tyr1158/Tyr1162/Tyr1163) antibody and detected by ECL. The nitrocellulose membrane was then stripped with buffer Restore and, subsequently, reprobed with an anti-IGF1R rabbit polyclonal antibody. The membranes were blotted with an anti-β-actin antibody to control for protein loading. Panel B: Densitometric analysis was performed on two independent experiments. Data are the mean ± s.e.m. of two independent experiments. *P < 0.05. CTL, control; P-IGF1R, phosphorylated IGF1R.

To confirm the results obtained by the real-time PCR, lymphocyte lysates underwent to SDS-PAGE to evaluate the IGF1R protein relative content and the IGF1R tyrosine kinase activity after stimulation with 10 nM IGF1 (Fig. 2).

After densitometric analysis and normalization by β-actin, we found that Patient 1 had a significantly higher IGF1R content (1.3 fold) than that of the control, Patient 2 had an IGF1R content similar to that of the control, whereas the IGF1R content was lower in Patient 3 compared to that of the control (Fig. 2A). After stimulation with 10 nM IGF1, IGF1R was activated in both Patients 1 and 2 (Fig. 2A), although in the Patient 1, due to the higher IGF1R content, with a major increase respect to the relative unstimulated control (Fig. 2B). As expected, the IGF1R autophosphorylation was undetectable in Patient 3 (Fig. 2B).

## Discussion

In the present study, we report two cases of chromosome 15q26.3 duplication and one case of ring chromosome 15 syndrome. Despite the genetic heterogeneity of these patients, we recognized some common clinical features, which became clearer after reviewing the already published cases.

### Chromosome 15q duplication

To our knowledge, only one case of a *de novo* chromosome 15q26.3 duplication has been reported to date (8). Two familial 15q26.3 duplications, respectively of 1.48 and 0.77 Mb, have recently been described (9). To understand the role that this specific breakpoint has in the clinical feature of the 15q overgrowth syndrome, we evaluated the *IGF1R* gene expression, the IGF1R biochemical activity, serum IGF1 levels and the clinical feature of Patients 1 and 2, respectively having a 568 kb pure and a 650 kb impure (Patient 2 had also a 600 16p11.2 kb deletion, this latter known as an autism susceptibility locus (10)) chromosome 15q26.3 duplication. These represent the smallest chromosome 15q duplications described so far.

A review of all cases of chromosome 15q duplication described up to now follows.

#### Chromosome 15q duplication: review of literature and prevalence of the clinical characteristics

The structural abnormalities of chromosome 15q are rare. To the best of our knowledge, up to now, 96 patients with chromosome 15q duplication have been reported in literature. Among these, 28 patients showed distal 15q tetrasomy due to a mosaicism or to a neocentromer marker chromosome (NMC) (2, 3, 11, 12, 13, 14, 15, 16, 17, 18, 19, 20, 21, 22, 23, 24, 25, 26, 27, 28, 29, 30) and the others had 15q distal trisomy (1, 2, 3, 4, 5, 8, 31, 32, 33, 34, 35, 36, 37, 38, 39, 40, 41, 42, 43, 44, 45, 46, 47, 48, 49, 50, 51, 52, 53, 54, 55, 56, 57, 58, 59). The duplication of 15q chromosome can be classified in pure and impure forms, based on the presence of another chromosome abnormality (e.g. a deletion) in addition to the duplication. The comparison of the clinical findings between patients with pure and impure 15q duplication did not show major phenotypic differences with the exception for the life-span that seems lower in patients with impure15q duplication (1, 5).

We carefully reviewed the clinical features of all published patients with chromosome 15q duplication to estimate the prevalence of each of them. Patients with unknown measures or reports with no sufficient clinical data available were excluded from this analysis. The prevalence of each feature is summarized in Table 1.

**Table 1:** Prevalence of the main clinical features in the patients described in this and in previously published studies (patients with chromosome 15q distal pure and impure duplication, chromosome 15q tetrasomy, 15q deletion syndrome and chromosome ring 15).

	**15q duplication**						**15q deletion**		
	15q pure duplication		15q impure duplication		15q tetrasomy	15q trisomy	Ring chr. 15 syndrome		15q deletion syndrome
	Patient 1	Literature	Patient 2	Literature	Literature	Literature	Patient 3	Literature^a^	Literature^b^
Mental retardation	+	100% (16/16)	Yes	97.6% (40/41)	100% (12/12)	97.8% (44/45)	Yes	95%	Yes
Developmental delay	+	93.8% (15/16)	Yes	97.3% (37/38)	17/17 (100%)	94.6% (35/37)	Yes	−	Yes
Defective speech capacity	+	87.5% (14/16)	Yes	68.8% (11/16)	50% (2/4)	82.1% (23/28)	No	39%	No
Overgrowth	+	45.0% (9/20)	No	44.9% (22/49)	52.9% (9/17)	42.3% (22/52)	No	−	No
IUGR/growth retardation	−	20. % (4/20)	No	22.4% (11/49)	11.8% (2/17)	25% (13/52)	Yes	100%	Yes
Cardiac malformations	−	33.3% (6/18)	Yes	50.0% (18/36)	16.7% (1/6)	47.9% (23/48)	No	30%	Yes
Kidneys malformations	−	38.9% (7/18)	No	14.3% (5/35)	33.3% (7/21)	15.6% (5/32)	No	−	No
Genital/gonadal abnormalities^d^	+	40.0% (2/5)	No	45.9% (17/37)	18.2% (2/11)	54.8% (17/31)	No	30%	Yes
Cryptorchidism	+	25.0% (1/4)	Not known^c^	57.1% (8/14)	0% (0/3)	60.0% (9/15)	/	30%	Yes
Sperm abnormalities	+	NR	Yes	NR	NR	NR	/	NR	NR
Facial abnormalities^e^	+	100.0% (19/19)	Yes	98.0% (50/51)	100% (15/15)	98.2% (54/55)	Yes	Yes	Yes
Hands abnormalities	+	92.9% (13/14)	No	70.3% (45/64)	95.6% (22/23)	65.4% (36/55)	Yes	Yes	Yes
Arachnodactyly	−	7.1% (1/14)	No	25.0% (16/64)	21.7% (5/23)	21.8% (12/55)	No	No	No
Café-au-lait spots	−	0% (0/12)	Yes	5.0% (1/20)	0% (0/17)	6.7% (1/15)	Yes	30%	No

a Features reported by Butler *et al*. (66); ^b^from OMIM database (http://omim.org/clinicalSynopsis/612626?highlight=15%20syndrome%20deletion); ^c^the patient was adopted; ^d^including cryptorchidism, hypoplasia of genitalia, hypospadias, congenital bilateral inguinal hernia, congenital hydrocele; ^e^including down-slanting palpebral features, micrognathia, low-set ears, high-arched palate, prominent nose, frontal bossing.

*Mental retardation* Mental retardation is a common feature in patients with 15q distal duplication. Patients 1 and 2 share with patients previously reported in literature neurological symptoms such as developmental delay, mental and psychomotor retardation, marked defective speech capacity (Table 1). As Chen and coworkers reported, the region 15q24.3-qter contains several genes involved in brain development and functioning (Table 2) (58). However, both Patients 1 and 2 had a chromosome 15q26.3 duplication, and none of these genes map in on this duplicated segment. Thus, neurological symptoms such as mental and psychomotor retardation, defective speech capacity and developmental delay found in the patients described in this study may be due to genes mapping on the chromosomal band 15q26.3.

**Table 2:** Genes involved in brain development and functioning located in the region 15q25.2–15q26.1.

**Gene name**	**OMIM**	**Mapping on**	**Encoding for**
*AP3B2*	602166	15q25.2	Adaptor-related protein complex 3, β2 subunit
*HOMER2*	604799	15q25.2	Homer homolog 2 (Drosophila)
*SH3GL3*	603362	15q25.2	SH3-domain GRB2-like 3
*NMB*	162340	15q25.2–q25.3	Neuromedin B
*CHD2*	602119	15q26.1	Chromodomain helicase DNA-binding protein 2

*Overgrowth and IUGR* Overgrowth is another common feature in patients with 15q distal duplication (3, 8, 12, 13, 16, 17, 23, 42, 48, 49, 51, 52, 54, 57, 59). Up to now, overgrowth has been attributed to the *IGF1R* gene overexpression (52).

Interestingly, IUGR and failure to thrive have also been described in these patients (2, 4, 5, 11, 18, 33, 35, 40, 43, 45, 46, 53, 55). Thus, triplication of the *IGF1R* gene does not seem to be sufficient to cause somatic overgrowth. Roggenbuck and coworkers hypothesized that the discordance genotype–phenotype may be attributed to the specific breakpoint, which may juxtapose the *IGF1R* gene next to a very active promoter or, alternatively, may remove it from its normal regulatory sequences (5). A similar explanation has also been given by other authors. Genesio and coworkers described the case of a multiple malformed female with a *de novo* inverted duplication of 15q (q21.3→26.3) associated with the deletion of the 15q telomere and part of the 15q26.3 band. She had a severe clinical course due to congenital heart defect, horseshoe kidney, hand contractures and clubfeet and death occurred after 20 days from birth because of cardio-respiratory failure. Curiously, she had marked IUGR. This feature was first ascribed to the 15q26.3 deletion, but FISH analysis revealed three copies of the *IGF1R* gene. Since this finding is not in agreement with the patient’s phenotype, the authors hypothesized that the clinical feature may depends on the global transcription dysregulation more than to the impairment of a single gene specifically correlated to the malformation (6). Finally, it has been also hypothesized that the discordance between the phenotype and the chromosome abnormalities may be explained by an hidden mosaicism, since failure to thrive has also been described in a 20-month-old female with a 24 Mb chromosome 15q25.1q26.3 duplication and mosaicism (2).

*Facial and skeletal abnormalities* Patients with chromosome 15q distal duplication commonly have facial abnormalities. The facial features more frequently reported are down-slanting palpebral fissures, prominent nose, low-set ears, micrognatia and high-arched palate. Long face, puffy cheeks, long philtrum, pointed chin and prominent occiput have also been reported (1, 3, 35, 40, 43, 49, 52, 59). In addition, they have hands abnormalities, which more frequently consist of arachnodactyly (Table 1), but syndactyly, clinodactyly, long fingers and joint contractures have also been reported (4, 5, 8, 31, 45, 52, 53).

*Cardiac malformation* Cardiac malformations have been found in about a half of patients (Table 1). These include patent ductus arteriosus, mitral valve stenosis, mitral valve arcade, patent foramen ovale, atrial and ventricular septum defect, cardiomegaly, atrio-ventricular canal, subaortic stenosis, Ebstein anomaly and Wolf–Parkinson–White (WPW) syndrome. Almost three genes related to the chromosome 15q duplication have been pointed out to be the potential cause of cardiac and vessel malformation: *ADAMTSL3* (OMIM 609199 – cytogenetic locations: 15q25.2) (29, 30, 60), *MESP1* (OMIM: 608689 – cytogenetic locations: 15q26.1) and *MESP2* (OMIM: 605195 – cytogenetic locations: 15q26.1) (29). *ADAMTSL3* gene overexpression has been proposed also to interfere with kidney function (60). This hypothesis is in line with the absence of a major cardiovascular or kidney malformation in patients 1 and 2 of the present study, since *ADAMTSL3*, *MEPS1* and *MEPS2* genes do not map in their duplicated region (15q26.3).

*Gonadal and genital abnormalities* Cryptorchidism (34, 38, 40, 43, 44) and genital abnormalities (including hypospadias, hypoplasia of external genitalia, congenital bilateral hernia and congenital hydrocele) (5, 25, 45, 51) have been reported in men with pure and impure chromosome 15q distal duplication. Unfortunately, it is noteworthy that the absence of a genital physical examination (e.g. no information on TV) and of gonadal function (e.g. serum LH, FSH and T levels) in the vast majority of the studies that we reviewed may underestimate the prevalence of gonadal abnormalities in these patients. Furthermore, no study has evaluated sperm parameters in these patients.

### Ring chromosome 15

Patient 3 had a ring chromosome 15 syndrome (46,XX r(15) (p10q26.2) karyotype) with a deletion of the terminal 5 Mb of chromosome 15. She had IUGR, developmental delay, mental and psychomotor retardation, café-au-lait spots, vitiligo and she underwent GH administration. Her clinical feature was similar to that of patients with a chromosome 15q26-qter deletion syndrome.

Jacobsen and coworkers were the first to describe the ring chromosome 15 syndrome and since then, about 40 cases have been reported in literature. Among these, only four cases were diagnosed prenatally (61). A ring chromosome origins from a breakage in both the arms of the chromosome and a fusion of the breakpoints, with consequent loss of the distal fragments. This results in monosomy of the distal short and long arms of the chromosome involved (7). In several cases of ring chromosome 15 syndrome, the *IGF1R* gene is deleted suggesting a role of IGF1R in the observed growth retardation (62, 63, 64, 65). In a review of 25 cases, the following main features were found: IUGR (100%), variable degree of mental retardation (95%), microcephaly (88%), hypertelorism (46%) and triangular facies (42%), delayed bone age (75%), brachydactyly (44%), speech delay (39%) frontal bossing (36%), anomalous ears (30%), café-au-lait spots (30%), cryptorchidism (30%), cardiac abnormalities (30%) (66). These findings are in line with those of the latest clinical reports. In addition, congenital diaphragmatic hernia (CDH) is another clinical feature described in these patients (7, 61, 67, 68).

The clinical features of chromosome 15q26-qter deletion syndrome have been already summarized (http://omim.org/clinicalSynopsis/612626?highlight=15%20syndrome%20deletion): these patients have short stature, low birth weight, failure to thrive, microcephaly, neurological symptoms (delayed psychomotor development and mental retardation), typical facial abnormalities (micrognathia, triangular facies, low-set ears, strabismus, blepharophimosis and broad nasal bridge), congenital cardiac anomalies (septal defects), male genitalia abnormalities (hypospadias, cryptorchidism and micropenis), hand and feet abnormalities (brachidactyly, absent or hypoplastic middle phalanges of hands, talipes equinovarus).

### Candidate genes

As it is simple to note, mental retardation, delayed psychomotor development, IUGR, failure to thrive, congenital cardiac defects, gonadal and genital abnormalities and some facial traits (micrognathia, low-set ears, broad nasal bridge) are described both in patients with chromosome 15q duplication and in patients with ring 15 chromosome syndrome. Consequently, these clinical features may arise not from a decreased or increased expression of genes mapping on chromosome 15q, but from an imbalanced expression of these genes.

Patients 1 and 2 of the present study, who share the 15q26.3 breakpoint, showed the same clinical findings commonly described in previously reported patients with 15q duplication (Table 1), who generally have more proximal breakpoint sites and longer duplications. Hence, the clinical findings of patients with chromosome 15q duplication might be reasonably due to the impaired expression of genes mapped in the 15q26.3 chromosomal band. In addition, all patients with a ring 15 chromosome syndrome have a deletion of the 15q26.3 chromosomal band. Thus, the candidate genes responsible for the clinical characteristic described both in patients with 15q duplication and in patients with ring 15 chromosome syndrome might map within the 15q26.3 band.

Interestingly, an OMIM database research shows that some of the genes mapped on this chromosomal band are involved in cardiac, skeletal and neurological abnormalities. In fact, the *MEF2A* gene (OMIM 600660) encodes for a protein that maintains the appropriate mitochondrial content and the cytoarchitectural integrity in the postnatal heart in mice (69) and seems to be responsible for cardiac abnormalities (coronary artery disease and myocardial infarction) in humans (70). Moreover, *MEF2A* has been suggested as a plausible candidate gene responsible for CDH (71). In addition, MEF2A mRNA is found in brain (72) and may play a role during nervous system development (73). Thus, *MEF2A* gene may be a candidate gene for cardiac and neurological abnormalities in patients with chromosome 15q duplication and ring 15 chromosome syndrome, and for CDH in patients with ring 15 chromosome syndrome.

*CHSY1* gene (OMIM 608183) encodes for the chondroitin sulfate synthase 1 that synthetizes a glycosaminoglycan expressed on the surface of most cells and in extracellular matrices. It causes the temtamy preaxial brachydactyly syndrome (73). Li and coworkers showed that in developing zebrafish, both loss and gain of *CHSY1* gene function lead to defects similar to those in human patients with temtamy preaxial brachydactyly syndrome, such as reduced body length, compromised formation of the pectoral fin, severe midline deficiencies in the cartilage of the neurocranium and compromised formation of the epithelial protrusions and semicircular canals in the inner ear (74). Hence, *CHSY1* might be a candidate gene responsible for skeletal abnormalities in patients with chromosome 15q duplication and ring 15 chromosome syndrome.

*MTR27* gene (OMIM 614340) has been reported to be responsible for delayed psychomotor development with very poor or lack of speech, head nodding, mild flattening of the midface and hypotonia (75). It might play a role in the incoming of neurological disorders in patients with 15q duplication and with ring 15 chromosome syndrome.

*IGF1R* gene may also be involved in the etiology of neurological symptoms of these patients. In fact, IGF1 protein has been reported to pass through the blood–brain barrier, and it is involved in normal central nervous system (CNS) development by promoting neuronal cell survival and synaptic maturation, thus facilitating functional plasticity in the brain (76). It has been hypothesized that elevated IGF1 levels in the cerebrospinal fluid may, in combination with a reduced *IGF1R* gene expression due to a chromosome 15q deletion or to a ring chromosome 15 syndrome, alter brain normal development (76).

None of the genes mapped in the chromosome 15q26.3 band seems to play a role in the etiology of gonadal abnormalities, apart from the *IGF1R* gene. A rational explanation of the presence of this feature has been hypothesized by Nef and coworkers (77). They reported that the insulin receptor (IR) tyrosine kinase family (comprising *IR*, *IGF1R* and IR-related receptor (*IRR*)) is required for the development of male gonads and thus for male sexual differentiation. In fact, XY mice that were mutant for all 3 receptors developed ovaries and showed a completely female phenotype. The decreased expression of both *Sry* gene and the early testis-specific marker Sox9 in these mice suggests that the insulin signaling pathway is required for male sex determination (77). In addition, they observed sex-reversed phenotypes only when both *IGF1R* alleles were mutated, while male embryos with one mutant allele for *IR* or no mutant alleles for *IRR* showed a partially sex-reversed phenotype. These data support the idea that a threshold of insulin family signaling is required to mediate normal male gonad differentiation: in particular, the overall contribution of *IGF1R* is crucial and higher than that of *IR*, which is itself higher than that of *IRR* (77).

Similar studies in human do not exist. The evaluation of the *IGF1R* gene expression in men with gonadal abnormalities would help to better understand the role that this gene might have in the development of male gonads and in male sex differentiation in humans.

Finally, as far as we know, the presence of café-au-lait spots has been hardly reported in patients with chromosome 15q duplications. We describe the case of a patient with an impure chromosome 15q duplication and café-au-lait spots. This feature has also been reported in 30% of patients with ring 15 chromosome (66). Although there is no evidence for a role of *IGF1R* on café-au-lait spot pathogenesis, some GH-excess syndromes (e.g. neurofibromatosis, McCune-Albright) include café-au-lait spots (78). On the other hand, café-au-lait spots might be ascribed also to the presence of the ring chromosome, apart from the specifically involved chromosome, as previously described (79). In the light of these evidences, further studies are needed to evaluate the possible role of the GHRH/GH/IGF1 axis, if any, in the etiopathogenesis of this sign.

The limitations of our study include the use of a 17-year-old male as control: although it could be a good choice for patients 1 and 2, its age and sex differ from those of patient 3. However, we think that the results found in this latter patient are reliable, because they fit well with the clinical case. Furthermore, the prevalence of gonadal and genital abnormalities (Table 1) may be underestimated, since the major of studies did not include the gonadal/genital evaluation at the physical examination.

## Conclusions

In conclusion, we found that the IGF1R mRNA and protein expression were elevated in a patient with a 568 kb 15q26.3 pure duplication, normal in a patient with an impure 15q26.3 650 kb duplication and a 16p11.2 600 kb deletion and decreased in a patient with ring chromosome 15. IGF1R function was normal in both patients 1 and 2 and undetectable in patient 3. Clinically, patient 1 had overgrowth, moderate mental and psychomotor retardation, marked defective speech, right hand cryptorchidism, severe oligoasthenoteratozoospermia, obesity, borderline low TV, borderline low serum T levels, gynecomastia and triangular distribution of pubic hair. Patient 2 had overgrowth, mild mental retardation, marked defective speech, café-au-lait patches, precocious puberty and severe oligoasthenoteratozoospermia. Finally, patient 3 had IUGR, failure to thrive, mental and psychomotor retard, café-au-lait patches.

We reviewed 96 patients with chromosome 15q duplication and we summarized the main clinical features in Table 1. We found that some clinical features of these patients, such as mental retardation, delayed psychomotor development, congenital cardiac defects, genitalia abnormalities and some facial traits are shared also by patients with ring 15 chromosome syndrome. We suggest that these common clinical features might be due both to the over and down expression of genes mapped on the chromosome 15q26.3.

We speculate that the *IGF1R* gene may play a role in the etiology of neurological disorders and of gonadal abnormalities in these patients. Accordingly, the relevance of IGF1R in the male gonads development has been already shown in mice (77). Further studies are necessary to evaluate the role that the *IGF1R* gene plays in the development of male gonads, in male sex differentiation and sperm abnormalities in humans.

## Declaration of interest

The authors declare that there is no conflict of interest that could be perceived as prejudicing the impartiality of the research reported.

## Funding

This research did not receive any specific grant from funding agencies in the public, commercial, or not-for-profit sectors.

## Authors’ contribution statement

Rossella Cannarella: Conception and design of the study, data acquisition, analysis and interpretation of data, drafting of the article, final approval of the version submitted. Teresa Mattina: Analysis and interpretation of data, drafting of the article, final approval of the version submitted. Rosita A Condorelli: Acquisition of data, analysis and interpretation of data, drafting of the article, final approval of the version submitted. Sandro La Vignera: Acquisition of data, analysis and interpretation of data, drafting of the article, final approval of the version submitted. Laura M Mongioì: Acquisition of data, analysis and interpretation of data, final approval of the version submitted, drafting of the article. Giuseppe Pandini: Acquisition of data, analysis and interpretation of data, drafting of the article, final approval of the version submitted. Aldo E Calogero: Conception and design of the study, analysis and interpretation of data, critical revision of the manuscript and final approval of the version submitted.
